# Lipotoxicity-Induced PRMT1 Exacerbates Mesangial Cell Apoptosis via Endoplasmic Reticulum Stress

**DOI:** 10.3390/ijms18071421

**Published:** 2017-07-03

**Authors:** Min-Jung Park, Ho Jae Han, Dong-il Kim

**Affiliations:** 1 Department of Molecular and Integrative Physiology, University of Michigan Medical School, Ann Arbor, MI 48109, USA; minjunp@med.umich.edu; 2 Department of Veterinary Physiology, College of Veterinary Medicine, Research Institute for Veterinary Science, Seoul National University, Seoul 08826, Korea; hjhan@snu.ac.kr; 3 BK21 PLUS Program for Creative Veterinary Science Research Center, Seoul National University, Seoul 08826, Korea; 4 Life Science Institutes, University of Michigan, Ann Arbor, MI 48109, USA

**Keywords:** PRMT1, lipotoxicity, mesangial cell, apoptosis, ER stress

## Abstract

Lipotoxicity-induced mesangial cell apoptosis is implicated in the exacerbation of diabetic nephropathy (DN). Protein arginine methyltransferases (PRMTs) have been known to regulate a variety of biological functions. Recently, it was reported that PRMT1 expression is increased in proximal tubule cells under diabetic conditions. However, their roles in mesangial cells remain unexplored. Thus, we examined the pathophysiological roles of PRMTs in mesangial cell apoptosis. Treatment with palmitate, which mimics cellular lipotoxicity, induced mesangial cell apoptosis via protein kinase RNA-like endoplasmic reticulum kinase (PERK) and ATF6-mediated endoplasmic reticulum (ER) stress signaling. Palmitate treatment increased PRMT1 expression and activity in mesangial cells as well. Moreover, palmitate-induced ER stress activation and mesangial cell apoptosis was diminished by PRMT1 knockdown. In the mice study, high fat diet-induced glomerular apoptosis was attenuated in PRMT1 haploinsufficient mice. Together, these results provide evidence that lipotoxicity-induced PRMT1 expression promotes ER stress-mediated mesangial cell apoptosis. Strategies to regulate PRMT1 expression or activity could be used to prevent the exacerbation of DN.

## 1. Introduction

Diabetic nephropathy (DN), one of the main causes of chronic kidney disease, is developed by 30–40% of diabetic patients, making it one of the most common complications of diabetes [1]. The development of DN is characterized by hypertrophy of the basement membrane of the glomerulus, which gradually becomes hypertrophic. Mesangial cells are the principal component of the glomerular mesangium, and, upon glomerular hypertrophy, the number of mesangial cells is increased, correlating with an increase in albumin excretion rate [2]. However, during the progression to overt nephropathy, mesangial cells are lost via apoptosis, leading to renal dysfunction [3,4]. Multiple pathogenic mechanisms have been implicated in the development of DN, including hyperglycemia, oxidative stress, cytokines, and genetic susceptibility [5]. One important mechanism implicated in DN is lipotoxicity or the accumulation of excess long-chain fatty acids in cells other than adipocytes [6,7]. It is known that mesangial cells are susceptible to lipotoxicity, and lipotoxicity-induced apoptosis of mesangial cells has been implicated in the development of renal failure [8,9]. However, the mechanism by which lipotoxicity induces apoptosis in these cells is not yet well understood.

Endoplasmic reticulum stress (ER stress) is a physiological and pathological response to the excessive accumulation of unfolded or misfolded proteins in the ER. ER stress has been implicated in mediating lipotoxicity-induced apoptosis in multiple cell types, including renal proximal cells [10,11]. The ER stress pathway is mediated through three major proteins, protein kinase RNA-like endoplasmic reticulum kinase (PERK), inositol-requiring enzyme 1 (IRE1), and activating transcription factor 6 (ATF6) [12]. PERK is activated by autophosphorylation in response to the detection of misfolded proteins; in turn, it phosphorylates eukaryotic inhibition factor α (eIF2a), leading to an increase in the expression of apoptotic genes such as CCAAT-enhancer binding protein homologous protein (CHOP). IRE1 is an endoribonuclease that, upon activation by autophosphorylation, carries out the unconventional splicing of X-box binding protein-1 (XBP1) mRNA. Finally, the proteolytic cleavage of ATF6 by S1P and S2P in the Golgi allows the cytoplasmic subunit p50ATF6 to translocate to the nucleus and act as a transcription factor.

Protein arginine methyltransferases (PRMTs) are enzymes that catalyze the methylation of arginine residues. In mammals, nine PRMTs (PRMT1-9) exist, and they are subdivided into three types according to the manner of methylation. Type I PRMTs (PRMT1, 2, 3, 4, 6, 8) catalyze the asymmetric dimethylation of substrates, while type II PRMTs (PRMT5, 9) catalyze symmetric dimethylation and type III PRMT (PRMT7) catalyzes monomethylation [13]. Increased PRMT1 expression in proximal tubule cells has been reported to be critical to the progression of diabetic nephropathy [14]. However, the regulatory and functional roles of PRMTs in mesangial cell apoptosis remain unexplored.

Previously, we reported that lipotoxicity is observed in mesangial cells under diabetic conditions [15]. We also recently reported that palmitate treatment, which mimics lipotoxicity, impairs hepatocyte function through the expression of PRMT1 and PRMT3 [16,17]. However, the role of PRMTs in mesangial cell apoptosis has not yet been evaluated. Thus, in this study, we have subjected cultured rat mesangial cells to a palmitate challenge and fed a high fat diet to mice to induce lipotoxicity in order to elucidate the role of PRMTs in lipotoxicity-induced mesangial cell apoptosis and the pathogenesis of diabetic nephropathy.

## 2. Results

### 2.1. Lipotoxicity Induces Mesangial Cell Apoptosis via PERK and ATF6 Signaling Pathways

**Figure 1:**
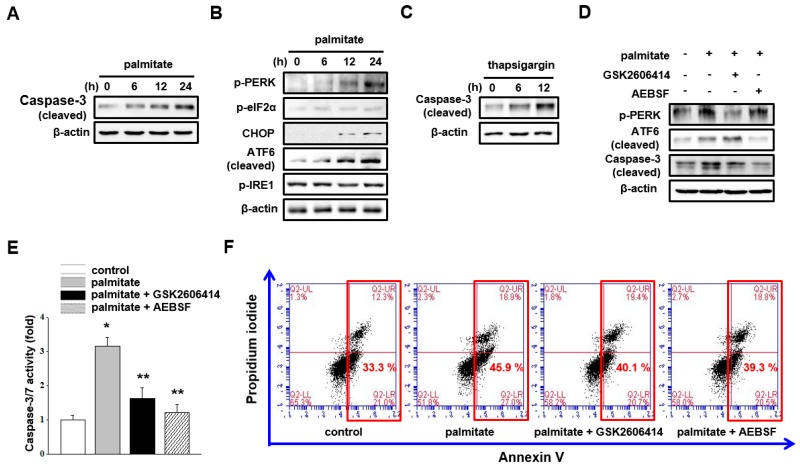
Lipotoxicity induces mesangial cell apoptosis via protein kinase RNA-like endoplasmic reticulum kinase (PERK) and ATF6 signaling pathways. (**A**,**B**) Mesangial cells were treated with 30 μM palmitate for various time intervals. Cell extracts were subjected to western blot analysis with indicated antibodies. The representative immunoblots were from at least three independent experiments; (**C**) mesangial cells were treated with 50 nM thapsigargin for 6 h and 12 h. Cell extracts were subjected to western blot analysis with caspase-3 antibody. The representative immunoblots were from at least three independent experiments; (**D**–**F**) mesangial cells were pretreated with 1 μM GSK2606414 or 100 μM AEBSF. After 30 min, cells were treated with 30 μM palmitate for 24 h; (**D**) cell extracts were subjected to western blot analysis with indicated antibodies. The representative immunoblots were from at least three independent experiments; (**E**) caspase 3/7 activity was measured. Data represent the mean ± SEM of three independent experiments, each performed in triplicate. * *p* < 0.05 vs. control, ** *p* < 0.05 vs. palmitate; (**F**) annexin V and propidium iodide stainings were analyzed. Red frame indicates the sum of early and late apoptotic cells. Representative images were from at least three independent experiments.

Palmitate-mediated lipotoxicity is known to induce mesangial cell apoptosis [8,9]. As expected, palmitate treatment increased caspase-3 cleavage in cultured mesangial cells (Figure 1A). Moreover, palmitate treatment activated PERK signaling (PERK phosphorylation, eIF2α phosphorylation, and CHOP expression) and ATF6 signaling pathways (ATF6 cleavage) but did not activate the IRE1 signaling pathway (Figure 1B). To confirm whether ER stress signaling activation is linked to mesangial cell apoptosis, mesangial cells were treated with thapsigargin, which triggers ER stress by inhibiting sarcoplasmic/ER Ca^2+^-ATPase (SERCA), leading to Ca^2+^ depletion. Thapsigargin treatment increased caspase-3 cleavage, suggesting that ER stress signaling is involved in mesangial cell apoptosis (Figure 1C). To further confirm, we employed the PERK inhibitor GSK2606414, which blocks PERK phosphorylation, and the S1P inhibitor AEBSF, which blocks ATF6 cleavage. As shown in Figure 1D, palmitate-induced caspase-3 cleavage was attenuated by GSK2606414 and AEBSF treatment. In addition, palmitate-induced caspase 3/7 activity and the increased number of apoptotic cells were diminished by treatment with the two inhibitors (Figure 1E,F). These results suggest that lipotoxicity induces mesangial cell apoptosis via PERK and ATF6 signaling pathways.

### 2.2. PRMT1 Expression Is Increased in Palmitate-Treated Mesangial Cells

**Figure 2:**
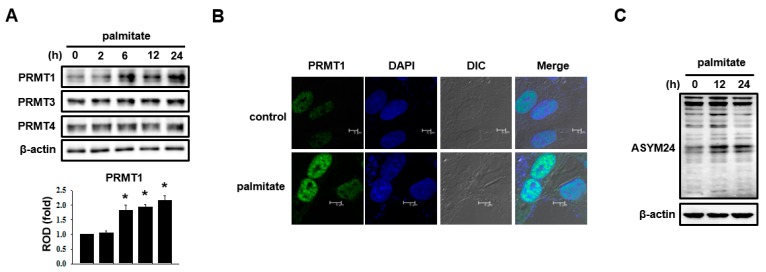
Protein arginine methyltransferase 1 (PRMT1) expression is increased in palmitate-treated mesangial cells. (**A**) Mesangial cells were treated with 30 μM palmitate for various time intervals. Type I PRMT expressions were assayed by western blot analysis. The representative immunoblots were from at least three independent experiments. Data represent the means ± SEM of three independent experiments. * *p* < 0.05 vs. 0 h (ROD: relative optical density); (**B**) mesangial cells were treated with 30 μM palmitate for 24 h. The cells were labeled with anti-PRMT1 and a fluorescein isothiocyanate (FITC)-conjugated secondary antibody. Nuclei were stained with 4′,6-diamidino-2-phenylindole (DAPI) and observed under a confocal microscope. The representative images were from at least three independent experiments (scale bar: 5 μm); (**C**) mesangial cells were treated with 30 μM palmitate for 12 h and 24 h. Cell extracts were subjected to western blot analysis with ASYM24 antibody. Representative immunoblots were from at least three independent experiments.

Type I PRMTs, which induce asymmetric dimethyl arginine generation, are closely related to the progression of diabetic nephropathy [18]. Mesangial cells in the glomerulus are crucial for the maintenance of a normal glomerular filtration rate. Nevertheless, the expression of type I PRMTs in mesangial cells under pathologic conditions has not been explored. Palmitate treatment increased PRMT1 expression, whereas the expression of PRMT3 and PRMT4, which are also type I PRMTs, was not altered (Figure 2A). Immunofluorescence studies also revealed that PRMT1 expression is increased by palmitate treatment and that its expression is observed predominantly in the nuclei of mesangial cells (Figure 2B). Moreover, PRMT1 activity, determined by western blotting using an antibody for ASYM24, which recognizes asymmetrically dimethylated arginine, was elevated by palmitate treatment (Figure 2C).

### 2.3. PRMT1 Knockdown Attenuates Palmitate-Induced ER Stress Signaling and Mesangial Cell Apoptosis

**Figure 3:**
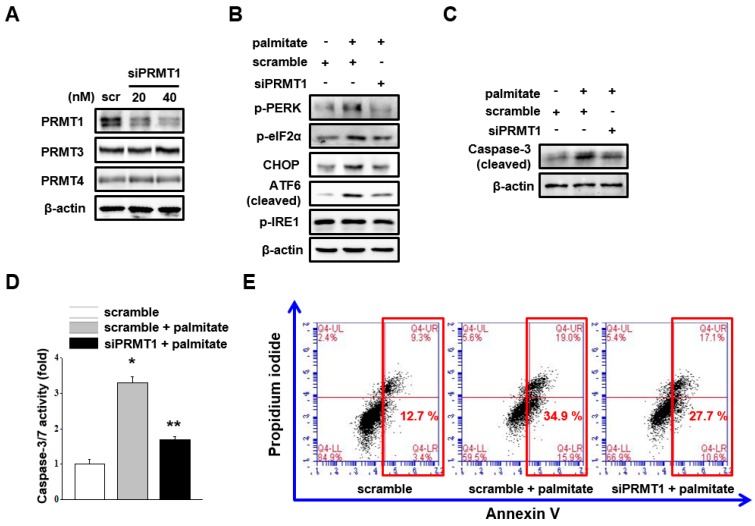
PRMT1 knockdown attenuates palmitate-induced ER stress signaling and mesangial cell apoptosis. (**A**) Mesangial cells were transfected with scramble or PRMT1 siRNA according to the reverse transfection method. After 36 h, cell extracts were subjected to western blot analysis with indicated antibodies. The representative immunoblots were from at least three independent experiments; (**B**–**E**) mesangial cells were transfected with scramble or PRMT1 siRNA according to the reverse transfection method. After 24 h, cells were treated with 30 μM palmitate for 24 h; (**B**,**C**) cell extracts were subjected to western blot analysis with indicated antibodies. Representative immunoblots were from at least four independent experiments; (**D**) caspase 3/7 activity was measured. Data represent the mean ± SEM of three independent experiments, each performed in triplicate. * *p* < 0.05 vs. scramble, ** *p* < 0.05 vs. scramble + palmitate; (**E**) annexin V and propidium iodide stainings were analyzed. Red frame indicates the sum of early apoptotic cells and late apoptotic cells. The representative images were from at least three independent experiments.

To determine whether palmitate-induced ER stress and apoptosis is due to elevated PRMT1 expression, endogenous PRMT1 was silenced by *prmt1* siRNA transfection without the alteration of other type I PRMTs. (Figure 3A). The knockdown of PRMT1 attenuated palmitate-induced ER stress signaling, except for IRE1 phosphorylation (Figure 3B). Moreover, palmitate-induced mesangial cell apoptosis was diminished by *prmt1* siRNA transfection (Figure 3C–E). These results suggest that PRMT1 mediates palmitate-induced ER stress activation and mesangial cell apoptosis.

### 2.4. HFD-Induced Glomerular Apoptosis Is Attenuated in PRMT1 Haploinsufficient Mice

**Figure 4:**
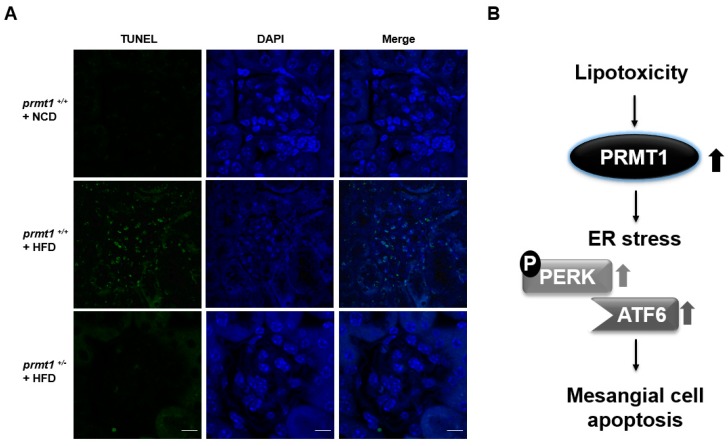
High fat diet (HFD)-induced glomerular apoptosis is attenuated in PRMT1 haploinsufficient mice. (**A**) Kidney sections were subjected to terminal deoxynucleotidyl transferase dUTP nick-end labeling (TUNEL) assay as described in “Materials and Methods”, (scale bar: 20 μM); (**B**) summarized scheme; lipotoxicity-induced PRMT1 exacerbates mesangial cell apoptosis via ER stress.

To confirm in vivo, we investigated the glomeruli of high fat diet (HFD) mice since HFD is known to contribute to intrarenal-lipotoxicity [8,19,20]. We used PRMT1 haploinsufficient (*prmt1*^+/−^) mice to evaluate the roles of PRMT1 in vivo as PRMT1 knockout mice are embryonically lethal [21]. The number of apoptotic cells, as determined by a terminal deoxynucleotidyl transferase dUTP nick-end labeling (TUNEL) assay, in the glomerulus was significantly elevated in mice fed HFD for 12 weeks (Figure 4A). However, HFD-induced glomerular apoptosis was significantly attenuated in *prmt1*^+/−^ mice compared with wild-type (WT; *prmt1*^+/+^) mice (Figure 4A), implying that PRMT1 could have a significant function in glomerular apoptosis.

## 3. Discussion

Lipotoxicity is one of the main causes of worsening DN. However, its precise mechanisms are not fully understood. In the present study, we determined that lipotoxicity-induced mesangial cell apoptosis is due to increased PRMT1 expression. Palmitate treatment significantly increased PRMT1 expression without the alteration of other type I PRMTs (PRMT3 and PRMT4). The subcellular distribution of PRMT1 is known to be cell-type specific. In human osteosarcoma cells (U2OS), PRMT1 was predominantly expressed in the cytoplasm, whereas it was primarily expressed in the nucleus in human breast adenocarcinoma cells (MCF-7) [22]. Using confocal microscopy, PRMT1 expression was mainly observed in the nucleus of mesangial cells but also slightly in the cytoplasm. This seems to suggest the possibility that PRMT1 acts as a transcriptional cofactor or methylates Histone 2A and Histone 4 to increase the transcription of target genes involved in ER stress and apoptosis in mesangial cells [23].

Palmitate-induced ER stress signaling was weakened by the knockdown of PRMT1. This result provides strong evidence that PRMT1 positively regulates ER stress. Recently, Liao et al. reported that PRMT1 can be localized in the ER lumen [24]. One can imagine that PRMT1 localized in the ER lumen could facilitate PERK phosphorylation and ATF6 cleavage. Further studies should be performed to reveal which part of PRMT1 (nuclear PRMT1 vs. ER lumen PRMT1) plays a role in ER stress signaling activation in mesangial cells.

In a previous study, we revealed that increased PRMT1 expression is associated with cellular apoptosis in lung epithelial cells and retinal pigment epithelial cells [25,26]. However, the roles of PRMT1 in apoptosis in other cells, especially non-tumor cells, were barely explored. The Fukamizu group revealed that the protein kinase B/Akt consensus sites (RxRxxS/T) for the BCL-2 antagonist of cell death (BAD) and Forkhead box O 1 (FOXO1) are methylated by PRMT1 and that methylation at this site competitively inhibits Akt-mediated phosphorylation [27,28]. In mesangial cells, the dephosphorylation of the BAD site is known to induce apoptosis [29]. Therefore, we anticipated that BAD would be a potent substrate of PRMT1 in lipotoxicity-induced mesangial cell apoptosis. However, we could not detect binding between PRMT1 and BAD using an immunoprecipitation assay in mesangial cells (Figure S1). In addition, we found that binding between PRMT1 and FOXO1 was unchanged by palmitate treatment in mesangial cells (Figure S1). These results imply that PRMT1-mediated mesangial cell apoptosis is probably regulated by a mechanism other than the methylation of BAD and FOXO1. Further studies should be performed to reveal the PRMT1 substrates that induce mesangial cell apoptosis.

In vivo studies using a PRMT1 haploinsufficient mouse model demonstrated that PRMT1 deficiency confers protective effects against HFD-induced glomerular apoptosis. This confirms that the results from cultured mesangial cells are reproducible in an in vivo animal model. The results from both the cultured cells and the animal model suggest that PRMT1 expression is involved in the progression of diabetic nephropathy.

## 4. Materials and Methods

### 4.1. Materials

Dulbecco’s Modified Eagle’s Medium (DMEM), Ham’s nutrient mixture F-12, and fetal bovine serum (FBS) were purchased from Life Technologies (Gibco BRL, Grand Island, NY, USA). Palmitate and 4-(2-aminoethyl)benzenesulfonyl fluoride hydrochloride (AEBSF) were obtained from Sigma-Aldrich (St. Louis, MO, USA). The palmitate was prepared as described previously [30]. Thapsigargin and GSK2606414 were purchased from Millipore (Billerica, MA, USA). p-IRE antibody (ab48187), ATF6 antibody (ab11909), PRMT1 antibody (ab3768), and PRMT4 antibody (ab110024) were purchased from Abcam (Cambridge, UK). caspase-3 antibody (#9662), p-eIF2α antibody (#9721), and CHOP antibody (#2895) were purchased from Cell Signaling Technology (Beverly, MA, USA). ASYM24 antibody (#07-414) was obtained from Millipore. β-actin antibody (sc-1616) and p-PERK antibody (sc-32577-R) were purchased from Santa Cruz Biotechnology (Santa Cruz, CA, USA). All reagents were of the highest purity commercially available.

### 4.2. Cell Culture

The rat mesangial cell line was obtained from the American Type Culture Collection (ATCC, Rockville, MD, USA). Cells were grown in DMEM/Ham’s F-12 (1:1) supplemented with 10% fetal bovine serum (FBS) at 37 °C in 5% CO_2_ in air. Stock cultures of cells were subcultured once a week (split ratio 1:8). Cells were grown to confluence in 60 mm dishes in DMEM/Ham’s F-12 with 15 mM 4-(2-hydroxyethyl)-1-piperazineethanesulfonic acid) (HEPES) buffer, 10% FBS, 5.5 mM glucose, 0.35% additional sodium bicarbonate, 2.5 mM l-glutamine, and 1% penicillin/streptomycin at 37 °C. The media was changed every other day. Passaged cells were plated to yield near-confluent cultures at the end of the experiments.

### 4.3. Protein Extraction and Western Blotting

Cell pellets were lysed in Mammalian Protein Extraction Reagent (M-PER) (Thermo Fisher Scientific, Waltham, MA, USA). Protease inhibitor cocktail (Sigma-Aldrich) and phosphatase inhibitor cocktail I + II (Sigma-Aldrich) were added, and then the proteins were extracted according to the manufacturer’s instructions. Western blotting was performed as described previously [17].

### 4.4. Caspase-3/7 Activity Assay

Caspase-3/7 activity was measured with the Caspase-Glo 3/7 Assay Kit (Promega, Madison, WI, USA). Mesangial cells were seeded in a white-walled 96-well plate and then treated with palmitate. After 24 h, the cells were processed according to the manufacture’s instruction. GloMax Microplate Reader (Promega) was used for luminescence analysis.

### 4.5. Annexin V/Propidium Iodide Staining

Annexin V/Propidium iodide staining was performed with a FITC Annexin V Apoptosis Detection Kit (BD Biosciences, Billerica, MA, USA) according to modified manufacturer’s instructions. Mesangial cells cultured in 6-well plates were washed with phosphate-buffered saline (PBS) and then filled with 1 mL of Accutase (Innovative Cell Technologies, San Diego, CA, USA). After two minutes, the plates were gently tapped to detach the cells. The resuspended cells were immediately washed twice with cold PBS and then resuspended in a pre-chilled binding buffer, which contained fluorescein isothiocyanate (FITC)-conjugated annexin V and propidium iodide (PI). After a seven minute incubation on ice in the dark, apoptotic cells were analyzed by flow cytometry (Accuri C6, BD Biosciences). FITC and PI double negative cells (LL; lower-left) were regarded as healthy cells. FITC-positive and PI-negative cells (LR; lower-right) were considered as early apoptotic cells, and FITC and PI double positive cells (UR; upper-right) were late apoptotic cells. FITC-negative and PI positive cells (UL; upper-left) were regarded as necrotic. Apoptotic cells were determined by the sum of early apoptotic cells and late apoptotic cells (LR + UR; red frame).

### 4.6. Immunofluorescence and Confocal Microscopy

The cells were washed twice in PBS and fixed for 10 min with 4% paraformaldehyde in PBS. After three washes in PBS, fixed cells were permeabilized with 0.2% Triton X-100. 1% bovine serum albumin (BSA) solution was used for blocking. The cells were incubated with PRMT1 antibody (dilution ratio—1:100) for 15 h at 4 °C. After three washes in PBS, the cells were incubated with anti-rabbit FITC secondary antibody (Sigma-Aldrich). Then the cells were mounted on slides and the nuclei were visualized with 4′,6-diamidino-2-phenylindole (DAPI) present in the ProLong Gold Antifade Mounting Medium (Invitrogen, Carlsbad, CA, USA). Immunofluorescence imaging was performed as described previously [17].

### 4.7. siRNA Transfection

siRNA for PRMT1 (Santa Cruz Biotechnology; sc-41069) and scrambled siRNA (Quiagen, Hilden, Germany) were used for silencing the endogenous PRMT1 expression. 40 nM of each siRNA was transfected into mesangial cells using Lipofectamin™ RNAiMAX reagent (Invitrogen), following the reverse transfection method, as instructed by the manufacturers.

### 4.8. Animal Experiments and TUNEL Assay

Animal experiments were performed in accordance with the “Guide for Animal Experiments” (Edited by the Korean Academy of Medical Sciences) and approved by the Institutional Animal Care and Use Committee (IACUC) of Seoul National University (SNU121119-3; 19 November 2012). PRMT1 haploinsufficiency mice were kindly provided by Seung-Hoi Koo (Department of Life Science, Korea University, Seoul, Korea). Twelve male C57BL/6J mice were separated into two groups and age matched; six PRMT1 haploinsufficiency mice were one group. The normal diet group was challenged with 10% kcal fat containing feed (Research Diets, NJ, USA; D12450B) and the high fat diet group was challenged with 60% kcal fat containing feed (Research Diets, NJ, USA; D12492) for 12 weeks. After 12 weeks, all the mice were sacrificed and the kidneys were extracted. To make a tissue slide, fixed kidneys in 10% neutral buffered formalin were embedded in paraffin and cut at a 5 μm thickness using a microtome. The kidney sections were deparaffinized with Histochoice, rehydrated with serial diluted ethanol, and washed with distilled water. Staining was performed using the DeadEnd™ Fluorometric TUNEL System (Promega) according to the manufacturer’s instructions. The stained tissues were mounted on slides, and the nuclei were visualized with 4′,6-diamidino-2-phenylindole (DAPI) present in the ProLong Gold Antifade Mounting Medium (Invitrogen) and observed using a fluorescence microscope (Eclipse Ni-U, Intensilight C-HGFI, Nikon, Japan).

### 4.9. Statistical Analysis

The results were expressed as the mean ± SEM. Data are representative of three or four independent experiments. For two group comparisons, a student *t* test was used, and for multiple comparisons, one-way ANOVA by SPSS (SPSS Inc., Chicago, IL, USA), followed by the Tukey post hoc test, was used. A value of *p* < 0.05 was considered significant.

## 5. Conclusions

We found that palmitate treatment increases PRMT1 expression. These increases regulate ER stress-mediated mesangial cell apoptosis (Figure 4B). Strategies to decrease PRMT1 expression or reduce its enzymatic activity could be used to prevent the exacerbation of diabetic nephropathy.

## Supplementary Materials

Supplementary materials can be found at www.mdpi.com/1422-0067/18/7/1421/s1.
