# Supplementary material for: Lipotoxicity-Induced PRMT1 Exacerbates Mesangial Cell Apoptosis via Endoplasmic Reticulum Stress
Source: Int J Mol Sci. 2017 Jul 3;18(7):1421. doi: 10.3390/ijms18071421 (PMC5535913; doi:10.3390/ijms18071421)
Supplement: Supplementary file 1 [file ijms-18-01421-s001.pdf]

Supplementary information

Co-immunoprecipitation analysis

Mesangial cells were lysed in non-denaturing lysis buffer (25mM Tris [pH 7.4], 150mM NaCl, 1% NP-40, 1mM EDTA, 5% glycerol, and protease inhibitor mixture). 800 μg of protein was incubated with PRMT1 antibody (Bethyl Laboratories, A300-722A) or rabbit IgG (Cell Signaling Technology, #2729) overnight at 4 °C, followed by incubation with Protein A-Agarose beads (Santa Cruz Biotechnology, sc-2001) for 4 h. The beads were washed five times with lysis buffer and boiled in loading buffer to elute bound proteins. Eluted proteins were analyzed by Western blotting. FOXO1 antibody (#2880) were purchased from Cell Signaling Technology and BAD antibody (ab32445) were purchased from Abcam.

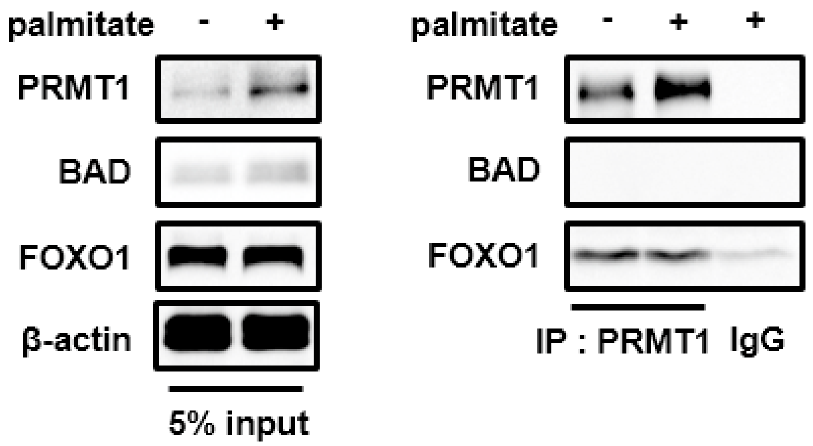

Figure S1. Mesangial cells were treated with 30 μM palmitate for 12 h. cell extracts were subjected to immunoprecipitation analysis with PRMT1 antibody as described in supplementary information. The representative immunoblots were from at least three independent experiments.
